# Impact and Intricacies of Bone Marrow Microenvironment in B-cell Lymphomas: From Biology to Therapy

**DOI:** 10.3390/ijms21030904

**Published:** 2020-01-30

**Authors:** Anuvrat Sircar, Sayan Mullick Chowdhury, Amber Hart, William Connor Bell, Satishkumar Singh, Lalit Sehgal, Narendranath Epperla

**Affiliations:** Division of Hematology, Department of Internal Medicine, The Ohio State University, Columbus, OH 43210, USA; sircar.4@buckeyemail.osu.edu (A.S.); Sayan.MullickChowdhury@osumc.edu (S.M.C.); hart.689@buckeyemail.osu.edu (A.H.); bell.1329@buckeyemail.osu.edu (W.C.B.); Satishkumar.Singh@osumc.edu (S.S.)

**Keywords:** diffuse large B-cell lymphoma, mantle cell lymphoma, follicular lymphoma, bone marrow microenvironment, relapse, drug resistance

## Abstract

Lymphoma, a group of widely prevalent hematological malignancies of lymphocyte origin, has become the focus of significant clinical research due to their high propensity for refractory/relapsed (R/R) disease, leading to poor prognostic outcomes. The complex molecular circuitry in lymphomas, especially in the aggressive phenotypes, has made it difficult to find a therapeutic option that can salvage R/R disease. Furthermore, the association of lymphomas with the Bone Marrow (BM) microenvironment has been found to portend worse outcomes in terms of heightened chances of relapse and acquired resistance to chemotherapy. This review assesses the current therapy options in three distinct types of lymphomas: diffuse large B-cell lymphoma, follicular lymphoma and mantle cell lymphoma. It also explores the role of the BM tumor microenvironment as a secure ‘niche’ for lymphoma cells to grow, proliferate and survive. It further evaluates potential mechanisms through which the tumor cells can establish molecular connections with the BM cells to provide pro-tumor benefits, and discusses putative therapeutic strategies for disrupting the BM-lymphoma cell communication.

## 1. Introduction

Lymphomas are a heterogeneous group of hematological malignancies that originate from a clonal proliferation of lymphocytes [1]. Despite a plethora of currently available treatment options, lymphomas have historically been a complicated disease in terms of clinical management, primarily due to their high prevalence of relapse [2]. From the initial classification into Hodgkin (HL) or non-Hodgkin lymphoma (NHL) based on the presence or absence of Reed-Sternberg cells, we have come a long way in our understanding of the biology and pathogenesis of lymphomas, as reflected in the most recent World Health Organization (W.H.O) classification which clarifies diagnosis of early-stage lymphomagenesis and elaborates on the genetic and molecular landscape of lymphoid neoplasms [3]. However, the question which still baffles clinicians and scientists alike, is why the currently available therapies often fail in some lymphoma patients in that they either do not respond or go on to lose response eventually. Furthermore, it is still not clear what provides these tumor cells with the cue to successfully abscond chemotherapy and if there is a valuable ’niche’ for these cells that can foster their growth and proliferation. The answers to these questions are thought to lie in the BM microenvironment. Most hematological malignancies including lymphomas have shown evidence of BM involvement at some point in their disease course [4,5,6,7]. Additionally, several studies have correlated BM involvement with worsened prognosis and impaired chemotherapeutic response in lymphomas [8,9,10]. This review delves into the current therapeutic options available for the treatment of B-cell non-Hodgkin lymphomas (specifically diffuse large B-cell lymphoma (DLBCL) as the most common aggressive subtype, follicular lymphoma (FL) as the most common indolent subtype and mantle cell lymphoma (MCL), which can have an aggressive or indolent course) and explores why and how the BM microenvironment proves to be a secure niche for lymphoma tumor cells. We specifically focus on these subtypes since they have varied BM involvement with 11%-34% of DLBCL cases [11,12], 70%-80% of FL cases [13] and 55%–90% of MCL cases [14] showing BM infiltration, respectively. Furthermore, these subtypes also represent both common and rare forms of NHL. We also discuss current strategies such as BM transplant and CAR-T cell (Chimeric antigen receptor (CAR) T-cell) therapy that can be used to overcome/reverse the BM derived survival cues and discuss potential strategies of effectively targeting the BM microenvironment to achieve higher therapeutic efficacy.

## 2. Current Therapeutic Landscape for Lymphoma

The therapeutic landscape for lymphoma treatment has ballooned in the last decade due to the emergence of new drugs and drug combinations that work through myriad mechanisms. The focus has particularly been heavy on treating R/R disease that continues to have dismal prognosis, especially in those with concomitant BM involvement [4,11,15]. However, the lack of randomized clinical trials in the R/R setting has led to minimal improvements in prognostic outcomes [16]. With the goal of garnering a better idea on the current therapeutic landscape, in this section, we summarize currently available treatment options for DLBCL, FL and MCL with a special focus on R/R disease.

DLBCL is the most common lymphoma and accounts for ~24% of new NHL cases annually [17]. Determination of cell of origin and molecular features of DLBCL is typically achieved through immunohistochemistry (IHC) staining [18] and fluorescence in situ hybridization (FISH) profiling [19,20], respectively. Based on IHC analysis DLBCL can be broadly divided into two subtypes: germinal center B-cell (GCB) and non-GCB subtypes [21]. IHC analysis also helps define the double expressor DLBCL (c-MYC/BCL-2 overexpression, 25% of de-novo DLBCL cases) [22,23], while FISH determines the double/triple hit lymphomas (genetic rearrangement of c-MYC/BCL-2 or BCL-6, 6-14% of de-novo DLBCL cases) [19,20]. Since the early 2000s, a multi-agent chemotherapy regimen involving rituximab, cyclophosphamide, doxorubicin, vincristine, and prednisone (R-CHOP) has been the cornerstone for the frontline treatment of DLBCL [24,25]. Although 50%–60% of DLBCL patients treated with R-CHOP achieve long-term disease-free survival, ~40%–50% will eventually relapse [26,27]. Patients with GCB DLBCL have a higher incidence of late relapses compared to non-GCB DLBCL [28]. Double expressor DLBCL which are responsible for ~50% of R/R DLBCL cases, have an intermediate response (~50%) to R-CHOP, whereas double/triple hit lymphomas have an extremely poor response [19,20,22,23,28]. Although efforts have been made to modify R-CHOP through the use of higher doses, longer more intensive treatment cycles and utilization of different agents (e.g., obinutuzumab in place of rituximab), such changes only provided a modest improvement in clinical outcomes [29,30,31].

In patients who fail to achieve remission with first-line therapy or relapse after having achieved complete remission, salvage chemotherapy followed by autologous hematopoietic stem cell transplantation (auto-HCT) is a putative option [32]. There are several salvage chemotherapy options including: rituximab, gemcitabine, dexamethasone and cisplatin (R-GDP); rituximab, etoposide, cisplatin, cytarabine, methylprednisolone (R-ESHAP); rituximab, ifosfamide, carboplatin and etoposide (R-ICE); and rituximab, dexamethasone, cytarabine and cisplatin (R-DHAP) with relatively similar efficacy rates but different toxicity profiles [33]. However, a majority of the relapsed patients fail to achieve responses with these salvage strategies [34]. The most common mechanism involves the proliferation of lymphoma cells that were resistant to the initial salvage chemotherapy. A less common mechanism involves the contamination of the autograft by lymphoma cells [35]. Of note, the outcomes of the relapsed patients vary depending on the timing of relapse with poor overall survival (OS) in those with primary refractory disease or early relapse compared to those with late relapses [36]. Newer therapies such as ibrutinib (a Bruton’s tyrosine kinase inhibitor which perturbs the B-cell receptor signaling cascade) as a standalone agent has been found to be minimally effective in R/R DLBCL, producing progression-free survival (PFS) of 1.7 months in GCB and three months in non-GCB, respectively [37]. Similar results were seen with lenalidomide as a single agent therapy with PFS of 7.8 weeks in GCB and 13.6 weeks in non-GCB, respectively [38]. However, R/R DLBCL patients who have been ruled ineligible for auto-HCT but have responded to a rituximab-based salvage chemotherapy regimen have been found to benefit from maintenance on lenalidomide and rituximab (R2) producing a 1-year PFS of 70% [39]. Bortezomib, a proteasome inhibitor has been found to be ineffective as a single therapy in R/R DLBCL. However, when combined with chemotherapy (Dose Adjusted Etoposide, Vincristine, Doxorubicin with Cyclophosphamide and Prednisone or DA-EPOCH) produces an overall response rate (ORR) of 93% [40]. Additionally, combination therapies such as Bendamustine plus Rituximab (BR) had an ORR of 17.5% (data presented at ASH 2017), while Nivolumab plus Ipilimumab (checkpoint inhibitors) produced an ORR of 20% [41] Other single-agent chemotherapeutic drugs of interest for R/R DLBCL are summarized under Table 1.

FL is the second most common lymphoma and is characterized by indolent disease biology. Around 26% of individuals are diagnosed at stage I (confined to a single region), while 27% of individuals are diagnosed at stage IV (diffuse or disseminated disease) [42]. FL is characterized by the reciprocal translocation t (14;18) (q32;q21), in which BCL-2 anti-apoptotic gene is placed under the control of the Immunoglobulin heavy chain gene rearrangement (IGH) associated transcriptional enhancers. The resulting overexpression of BCL-2 contributes to the evasion of apoptosis [43,44,45]. The gain of function mutations in the histone H3K27 methyltransferase are also common in FL [46]. The frontline treatment for FL is an anti-CD20 monoclonal antibody (rituximab or obinutuzumab) with chemotherapy (such as CHOP, CVP [Cyclophosphamide, Vincristine, Prednisolone] or Bendamustine [47]. More recently, non-chemotherapy options (rituximab and lenalidomide (R2)) have shown comparable efficacy in carefully selected patients [48]. More than 95% of newly diagnosed patients with FL will respond to front-line chemoimmunotherapy with a median OS of approximately 14 years [49].

Patients with FL (stage II and above) have a relapse rate of approximately 20% [49]. Most patients with R/R disease can be salvaged with chemoimmunotherapy or radioimmunotherapy approaches [50,51]. Patients who relapse/progress within 24 months of the diagnosis (POD24) have worse outcomes compared to those who relapse later [52]. A recent Center for International Blood and Marrow Transplant Research (CIBMTR) study showed that those who experience early treatment failure (ETF) after front-line chemoimmunotherapy may benefit from auto-HCT within one year of treatment failure [53]. Although chemoimmunotherapy regimens with fewer toxic effects have shifted the clinical focus away from auto-HCT as a routine treatment option in relapsed FL, none have demonstrated the potential for a cure [50]. Other key options at relapse include immunomodulators (R2, if not administered frontline) and PI3K inhibitors (such as idelalisib, copanlisib and duvelisib) [54] (Table 1). EZH2 inhibitors such as Tazemetostat [55] and BCL2 inhibitors such as venetoclax are also being evaluated as plausible therapy options, with promising results [56] (Table 1).

MCL is a relatively rare subtype of NHL that constitutes 5%–6% of all NHL [57]. The survival outcomes for MCL have improved relative to the past decade with a median overall OS of around 10 years post-detection [58]. The treatment for MCL is stratified based on age and transplant eligibility. The broad outline of treatment for “younger, fit” patients (fit to receive auto-HCT) involves the use of more intense potentially more toxic therapies that typically include induction therapy (R-Ara-C based chemotherapy) followed by consolidative auto-HCT and maintenance therapy (with single-agent rituximab) [59,60,61,62]. In elderly or “unfit” patients (unfit to receive auto-HCT) in whom control of disease progression is typically prioritized, therapy generally involves R-CHOP, BR, R-BAC500 (R-B-low dose Cytarabine) or VR-CAP (Bortezomib, Rituximab, Cyclophosphamide, Doxorubicin, Prednisone) regimens [63,64,65,66]. In very elderly “frail” patients, the primary goals of myelotoxicity prevention and tolerance to the treatment take precedence. Treatment options include R-chlorambucil, R with low dose bendamustine or R2 [67]. The overall response rates are >90% for patients receiving auto-HCT and >85% for those not receiving auto-HCT. Additionally, there are several ongoing trials incorporating novel agents in the frontline treatment for MCL.

Therapy for relapsed MCL patients primarily revolves around the use of BTK inhibitors such as ibrutinib [68] (Table 1), or acalabrutinib (which has greater selectivity and fewer off-target effects compared to ibrutinib) [69]. Other agents that are effective include R2 [70,71,72,73], Bortezomib [74,75], Temsirolimus [76,77,78] and Venetoclax [79] (Table 1). Chemotherapy-free treatment options are also being evaluated in relapsed MCL, primarily with R2 combinations.

## 3. Lymphoma and the BM Microenvironment

DLBCL, FL and MCL have distinct patterns of BM involvement. DLBCL shows a mixed pattern of BM involvement that can potentially range from localized focal infiltrates to complete replacement of BM with lymphoma cells [84]. In contrast, FL infiltration is primarily localized to the paratrabecular regions [84]. MCL primarily infiltrates paratrabecular regions as well, but has also shown evidence of focal infiltration [84]. The vast involvement of the BM tumor microenvironment in conferring pro-tumor survival benefits to lymphoma cells has been implicated to involve a variety of cell to cell ’communication signals’ between the BM components and the tumor. The human BM consists of a network of hematopoietic stem cells and lymphoid tissue that among other things are responsible for the synthesis of immune cells [78]. The intricate cross-talk between several other types of cells such as osteoblasts (which provide support for primitive hematopoietic cells), perivascular cells, endothelial cells (which aid production of growth factors and cytokines), adipocytes and macrophages lend further complexity to the BM microenvironment [85]. Additionally, the BM is also a residence for myeloid cells such as granulocytes, monocytes, dendritic cells, osteoclasts, erythrocytes and megakaryocytes [86]. Furthermore, stromal cells that can actively interact with lymphoma tumor cells have been found to play a key role in transforming the BM environment to aid lymphoma growth [87,88]. Common examples of such stromal cells include mesenchymal stromal cells (MSC’s), lymphoma associated macrophages (LAM), myeloid-derived suppressor cells (MDSC’s) and dendritic cells. MSC’s are known regulators of cellular proliferation and tissue differentiation. They are found throughout the body but within the BM they play the role of a key intermediary between tumor cells and the body’s immune responses [89]. Lymphoma cells co-injected with MSCs have shown evidence of decreased apoptosis, indicating that there may be an immunosuppressive component resulting from lymphoma cell-stromal cell interaction [88]. Enhanced recruitment of MSCs has been found to be facilitated by the chemokine CXC-chemokine ligand 12 (CXCL12) produced by tumor cells within the BM microenvironment [90]. A large number of CD14+ HLA-DR monocytic MDSCs, frequently found along with tumor cells has also been correlated with poor prognosis in DLBCL [91]. Interactions between lymphoma cells and MDSCs were found to result in reduced HLA-DR expression in patients, which is an indicator of immune suppression and an increasingly aggressive malignant tumor [92]. High levels of CD68+ and CD 163+ LAM in FL could be correlated to poor outcomes in conventional chemotherapy regimens before the advent of rituximab [93]. Similarly, direct dendritic cell contact in MCL has also been shown to prevent apoptosis in the tumor cells.

In terms of molecular mechanisms at play, multiple interconnected pathways functioning within the BM microenvironment have been found to influence and support lymphoma cell growth. Stroma and stromal cell directed activation of a variety of kinases on the surface of tumor cells has been shown to be a critical pathway leading to MCL survival and progression. For example, stroma based activation of focal adhesion kinase (FAK) has been shown to further activate other kinases in MCL, leading to a proliferation signaling cascade activation [94]. Furthermore, Sox11+ MCL cells have been demonstrated to exhibit greater adhesion to stromal cells and enhanced resistance to chemotherapy. Additionally, activation of CXCR4, which is involved in inducing tumor cell adhesion to stromal cells, has been shown to stimulate autophagy and influence greater survival of MCL cells [95]. There is also evidence of a ’dual benefit effect’ that human BM derived mesenchymal stem cells (hMSCs) can confer in DLBCL by the secretion of IL-6 and upregulation of IL-17A, simultaneously promoting both proliferation and drug resistance [9]. Lymphoma-stromal cell adhesion mediated increase in B-cell activating factor (BAFF) has also been reported to decrease apoptosis in response to chemotherapy [96].

**Figure 1:**
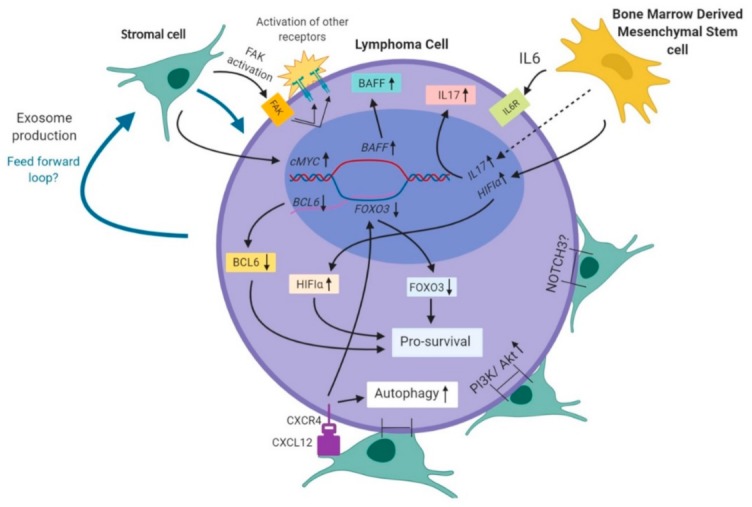
Suggested pathways for the promotion of tumor survival with the aid of the bone marrow microenvironment. Blue compartment: Nucleus, Purple compartment: Cytoplasm, Cells in green: stromal cells, Cells in yellow: BM derived mesenchymal stem cell. Text in black within the nucleus indicates the transcriptional change of respective components/genes. The subsequent effect on respective protein production is indicated through arrows outside the nucleus in the cytoplasm (names of proteins in boxes).

Given these observations, there is extensive ongoing research looking to validate the presence of a complex multifactorial autocrine/paracrine loop in the BM niche, whereby the tumor cells can direct the BM cells to modulate levels of pro-and anti-survival factors in order to favor its growth. For example, upregulated Notch-3 is a factor implicated in the development of aggressive lymphoma cells in the DLBCL-stromal cell co-culture system [97]. Abundant expression of Jun in DLBCL is responsible for tumor cell interaction with the BM stromal cells [98]. Positive expression of the PRC2, H3K27me3, and c-myc in BM microenvironment cells of DLBCL patients with BM involvement can be correlated with decreased OS [99]. While stromal cells act to primarily assist in tumor cell survival, immune cells such as cytotoxic T cells and regulatory T cells take a more active role in influencing tumor survival in the BM microenvironment. The presence of cytotoxic T cells (positive for PD-1) that can counteract the tumor cells were shown to be important factors that lead to positive prognosis in FL [100]. Similar findings in other forms of lymphoma underscore the justification for CAR-T cell-based therapy that is currently being explored for the treatment of R/R disease. In contrast, follicular helper T cells, which are found abundantly in FL have been found to positively influence lymphoma growth through STAT-5 signaling by providing IL-4 stimulation to the proliferating B cells. Similarly, suppressor T-regs, a type of regulatory T-cell that can suppress the activity of other immune cells such as natural killer cells, B-cells and bystander T-cells [101] have been found to be recruited by malignant B cells and hence over-represented in various NHL when compared to healthy patients [102]. The resultant suppression of immune cell function can potentially lead to a worsened prognosis in lymphoma patients. However, an increase in other forms of T-regs (Fox3+ cells) can also potentially contribute to improved immune surveillance and has been shown to reduce cell proliferation in FL and DLBCL, leading to improved treatment outcomes [100,103]. Overall, it is clear that several close interactions with stromal and immune cells within the BM in addition to modulation of chemokines and molecular pathway intermediates directly influence growth of DLBCL, FL and MCL, providing evidence that the BM niche plays a critical role in both lymphoma survival and drug resistance. Figure 1 summarizes the putative molecular mechanisms that can potentially contribute to lymphoma cell survival in the BM microenvironment.

## 4. BM Transplant in Lymphoma Treatment

**Figure 2:**
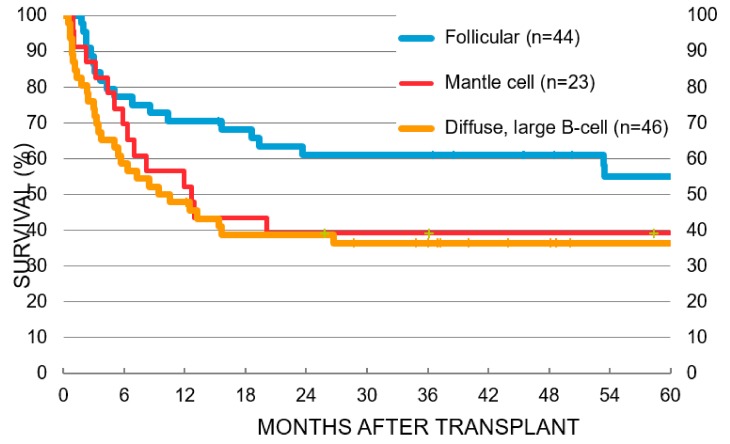
Data from the Center for International Blood and Marrow Transplant Research (CIBMTR) showing survival after first allo-HCT in FL, MCL and DLBCL patients. Reproduced with permission from the National Marrow Donor Program (NMDP).

As discussed in the previous section, a classical feature of most lymphomas is the involvement of the BM at some point in time during the disease course with the BM components playing crucial roles in aiding their growth and proliferation. As such, HCT has become a fairly common therapeutic option in DLBCL, FL and MCL, especially in R/R disease. HCT can be broadly classified into two types depending on the source of stem cells, auto-HCT (autologous, patient-derived) and allo-HCT (allogenic, donor-derived) [104]. Auto-HCT is an integral component of frontline treatment in MCL with improved outcomes when employed in CR1 (Complete Remission 1), although there are no randomized data comparing auto-HCT vs. no auto-HCT in CR1 at this time. In DLBCL, auto-HCT is commonly employed in CR2, with no clinical benefit observed when used in CR1 [105]. Lastly, in FL, auto-HCT is recommended in CR3 [106]. It is important to note however those patients who relapse following auto-HCT have poor OS following auto-HCT relapse. For instance, although, auto-HCT can salvage patients with relapsed DLBCL, ~50% of these patients will eventually relapse again with a poor OS following the relapse, especially in those who relapse within six months [107,108]. Similarly, early relapse following auto-HCT portends worse outcomes in patients with MCL [109]. While allo-HCT can potentially salvage patients who progress following auto-HCT [110], the outcomes of patients relapsing early still continue to remain poor [110]. Figure 2 shows the 5-year survival data obtained from CIMBTR for DLBCL, FL and MCL patients that have undergone allo-HCT without a previous auto-HCT. Results indicate that FL patients have ~55% survival, while MCL and DLBCL patients have ~40% and ~38% survival, respectively, after five years. Of note, haploidentical HCT (haplo-HCT) has changed the landscape of allo-HCT. In a CIBMTR study, the three year PFS of haplo-HCT was found to be 44% in DLBCL (*n* = 66), 66% in FL (*n* = 28) & 32% in MCL (*n* = 21) [111]. Another CIBMTR study that looked at the comparative outcomes after haplo-HCT using post-transplant cyclophosphamide to HLA-matched sibling donors, showed similar outcomes. There was no difference in the non-relapse mortality, progression/relapse, PFS or OS between haplo-HCT using PT-Cy and MSD allo-HCT [112]. Thus, haplo-HCT is a reasonable option for patients when a matched BM donor is not available.

The relative differences between the safety profile of allo-HCT and auto-HCT are also an important consideration to note, especially in terms of quality of life. Quality of life of HCT patients is subjective and not many studies have been done on this topic. However, as HCT becomes more efficient and common this will be an important factor in patient satisfaction and lifestyle. In general, auto-HCT is considered significantly safer than allo-HCT. A 2019 study that explored the overall health effects of patients following auto-HCT [113] determined that 41% of patients had no severe impairment of the tested domains (mobility, self-care, usual activities, pain/discomfort, anxiety/depression) while only 2% had all five impairments [113]. In contrast, allo-HCT has significant treatment-related mortality associated with it [114]. While auto-HCT has less of a chance of complications compared to allo-CT, it still may not be the treatment that works for patients and allo-HCT may be necessary eventually [115].

## 5. CAR-T Cell Therapy for Lymphoma Treatment

CAR-T cell therapy which involves expression of modified receptors on T cells to target tumor cell surface antigens has shown promise in lymphoma therapy in terms of successfully producing relatively long durations of complete remission in R/R lymphoma patients [116,117]. Currently, CD-19 targeting CAR-T cells are the only ones that are approved for clinical use. CD-19 is expressed ubiquitously on normal and neoplastic B-cells [118,119] while being completely absent on pluripotent BM stem cells [120]. As such, significant toxicity in the BM can be potentially avoided with this treatment modality while specifically targeting proliferating B cells within the BM. Yescarta (Axicabtagene ciloleucel) and Kymriah (Tisagenlecleucel) have been recently approved by the FDA for the treatment of patients with R/R DLBCL who have had two prior lines of therapy [121]. Tisagenlecleucel has also been reported to have produced an overall response rate of 53% in FL based on data of 24 patients from the JULIET trial [122]. ZUMA-2 trial with Axicabtagene ciloleucel for patients with R/R MCL has recently shown an overall response rate of 93% in a phase 2 trial [123]. Lisocabtagene maraleucel (anti CD-19) is another therapy currently under exploration (TRANSCEND trial) that has produced an overall response rate of 73% and complete remission of 43% in phase 1 trials thus far in DLBCL, transformed DLBCL and FL patients [124]. Table 2 summarizes the results from current CD-19 CAR-T cell based clinical trials currently underway for NHL patients. Overall, these results indicate that CAR-T cells are highly effective in treating R/R DLBCL, FL and MCL, and need to await long-term follow-up data to see the durability of this approach.

## 6. Newer Therapies Targeting BM Microenvironment

With the success of ibrutinib and related BTK inhibitors in NHL, especially in R/R MCL, it is worthwhile to look into potential mechanisms of interaction of ibrutinib with the tumor microenvironment, especially in the BM. One emerging hypothesis is that ibrutinib disrupts the molecular signaling cascades in the BM-tumor microenvironment and also reduces T-cell pseudo-exhaustion [126]. This could provide an explanation for why drugs like ibrutinib result in favorable OS in several lymphomas as compared to other single-agent drugs [127,128]. However, developing agents that can specifically target therapeutic agents into the BM and disrupt BM communication with high efficacy without producing non-specific systemic toxicity has been a challenge and is an area that needs more investigation. Towards that goal, ligand targeting CAR therapy is in the early stages of development in hematological malignancies with BM involvement [129]. However, natural ligands such as CAR antigen-binding domains may require further engineering to promote optimal binding and multimerization to adequately trigger T-cell activation.

Bispecific antibodies, which are engineered antibodies that can bind two unique epitopes and have a mechanism of action that is analogous to CAR-T cells and has the potential to improve therapeutic efficacy while decreasing non-specific toxicity in the BM [130,131]. Blinatumomab, a bi-specific antibody targeting CD19/CD3 has shown good efficacy in R/R NHL [132]. More recently, the data on the CD20/CD3 T-cell engaging bispecific antibodies especially mosunetuzumab has shown impressive activity in R/R B-cell NHL, including those who are refractory or relapsed following CAR-T cell therapy [133]. The preliminary data is very promising and if the results hold in validation studies, then this will provide another important treatment option, especially in those who fail to respond to CAR-T cell therapy.

**Figure 3:**
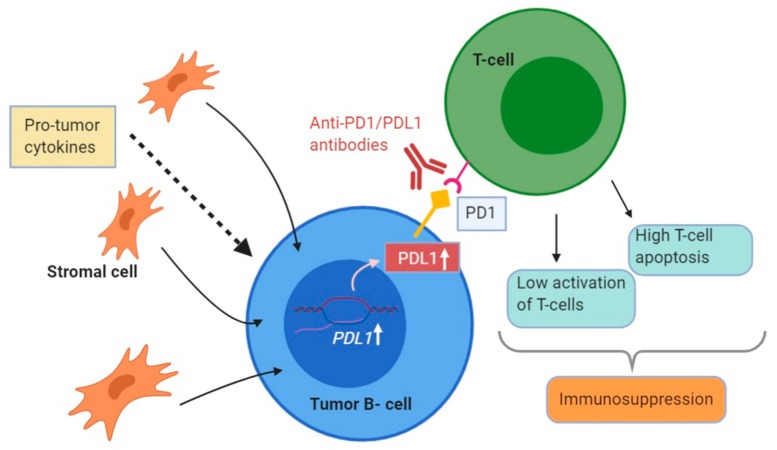
Schematic showing how the BM microenvironment can enhance PD-L1 expression on tumor cells, consequently leading to lower activation, greater apoptosis of T cells and higher immunosuppression.

Checkpoint blockade is another potentially effective strategy that is currently being studied for NHL therapy both pre and post CAR-T cell therapy. Checkpoint inhibitors function by restoring a cytokine and chemokine signaling balance in the BM microenvironment, effectively leading to improved immune surveillance and eradication of lymphoma cells that could previously escape the immune system. Monoclonal antibodies against PD-1/PDL-1 (Programmed Death-1/Programmed Death Ligand-1) and CTLA-4 (Cytotoxic T lymphocyte associated antigen 4) are the most studied therapies in this class. PD-L1 binding has also been studied in B-cell lymphomas as well as other hematological malignancies and can be a potential target for BM intervention to halt disease progression [134]. Binding of PD-L1 to PD-1 reduces cytokine production and activation of the target T cells, leading to an immunosuppressive microenvironment [134] (Figure 3). In vitro analysis has shown that pro-tumor cytokines and BM stromal cells increase PD-L1 expression on multiple myeloma cells, indicating that the BM microenvironment may play a role in the activation of the PD-1/PD-L1 pathway [135]. Clinical trials targeting the PD-1/PD-L1 pathway in B-cell NHLs provided modest clinical activity when used as a single agent (PD-1 inhibitors). Hence, using a combinatorial approach targeting the PD-1/PD-L1 pathway along with BCR signaling pathway inhibitors and or DNA damaging agents (cytotoxic chemotherapies) is the way moving forward. Currently, anti-PD-1 monoclonal antibodies being studied include Pidilizumab, Nivolumab and Pembrolizumab while anti-PDL-1 antibodies include Atezolizumab, Urelumab and Durvalumab. CTLA-4 is a con-inhibitory receptor found in the cytoplasm on naïve T cells which are transported to the T cell surface to bind ligands (CD80, CD86) and cause T cell downregulation [136]. As such, CTLA-4 inhibition potentially prevents T-cell downregulation and improves immune surveillance in the BM microenvironment. Ipilimumab is a monoclonal antibody currently being used as a CTLA-4 inhibitor in B-Cell Lymphomas.

Another therapeutic avenue to potentially explore is the reversal of hypoxic microenvironment in lymphoma. Hypoxic tumors, in general, have been found to be resistant to both chemotherapy and radiation and a hypoxic microenvironment has been found to promote resistance in leukemia-initiating cells, suggesting that hypoxia could be a therapeutic target in lymphomas as well [137]. Hypoxia has been found to increase the expression of CXCR4, consequently increasing the migration and homing of circulating cells to new BM niches [138]. One specific target of interest for studying hypoxia is the protein HIF1α which is stabilized post-transcriptionally by levels of oxygen tension less than 2%, making it overexpressed in a variety of tumors [138]. This idea has been heavily studied in multiple myeloma, where studies have suggested that hypoxia activates Epithelial Mesenchymal-Transition related machinery that decreases expression of E-cadherin and limits adhesion of multiple myeloma cells to the BM [137]. Importantly, HIF1α has also been found to be upregulated in lymphomas, especially in DLBCL [139]. As such, targeting the hypoxic microenvironment through modulation of HIF1α could be an effective target in limiting BM involvement in lymphoma growth.

While there are only a few studies exploring therapies that target the BM microenvironment specifically in lymphoma, similarities between the leukemic BM microenvironment and the lymphoma BM microenvironment can lead us to explore potential targets for niche-directed therapies that have been used to eradicate survival advantage and overcome drug resistance mediated through leukemic stromal cells. For example, significant deregulation of adhesion molecules that serve as interaction sites for direct cell-to-cell contact in BM has been useful in targeting leukemia cells and could potentially be useful in lymphoma [140].

Another mechanism that warrants investigation is the CXCR4-CXCL12 signaling abrogation that has been successful in treating acute myeloid leukemia. The CXCR4-CXCL12 axis is an essential regulatory pathway that mediates the interactions between the leukemic and BM niche cells, directs migration of leukemic cells into the BM and has also been implicated in chemoresistance of leukemia cells [137]. Dysregulated CXCR4 expression has already been correlated with lymphoma cell survival and often associated with a worsened prognosis in lymphoma patients [141]. As such the CXCR4-CXCL12 could be an appropriate target limiting BM mediated support of lymphoma growth in future studies. Targeting the BM microenvironment as a treatment strategy for lymphoma is a relatively new concept that is still largely being investigated. With several novel therapies that are currently being explored, it is also prudent to divert our attention to how the BM is adapting to these therapies over time. Current high dose chemotherapy has been found to deplete most of the resident BM cells and induce hematopoietic stem cell apoptosis and senescence, causing varying levels of myelosuppression and myelotoxicity which can often be irreversible [142]. The BM stroma, despite being more resistant to chemotherapy, is an essential component of the BM that contributes to repopulation and rejuvenation of the marrow once therapy is complete [142]. As such, treatment-induced complete depletion of the BM stroma or irreversible damage can potentially be detrimental to lymphoma patients in the long run. It will, therefore, be essential to focus our attention on strategies that can specifically target the lymphoma cells at the molecular level and cause minimum damage to other essential components of the BM. The development of resistance to therapies can also be an important consequence of adaptation strategies employed by the hematological malignancy infiltrated BM [10,143]. Such adaptation typically occurs by modifying cellular communication at the molecular level to induce the production of pro-survival cytokines and signaling intermediates that benefit the malignant cells [143]. Exploration of novel strategies that can circumvent such adaptive mechanisms will be critically important for establishing efficacious BM targeting treatments for lymphoma.

## 7. Conclusions

Extensive evidence from a plethora of research studies points to the fact that lymphoma makes prudent use of the BM microenvironment to gain pro-survival benefits and utilize the environment as a secure residence to avoid the chemotherapeutic strategies used against it. Hence, what starts as B-cell transformation eventually transforms into a ’parasitic’ tumor, feeding off the host BM resources to strengthen itself. As such, in the future, therapies that target and cut off the supply of factors facilitating tumor growth in the BM microenvironment are more likely to be effective (alone or in a combinatorial fashion), especially as a strategy for treatment in cases of R/R tumors which traditionally portend a poor prognosis. While many of these factors are secreted throughout the body, the BM microenvironment provides a concentrated niche of the supply of these nutrients for the cancer cells. This makes targeted delivery of the therapeutic agents to the BM microenvironment an important focus area that needs further exploration. A host of probable signaling factors have already been implicated, encompassing crosstalk between lymphoma cells, stromal cells, BM mesenchymal stem cells and other members constituting the complicated BM niche. Therefore, it would be prudent to direct our focus towards suppressing the release of pro-tumor factors to essentially ’reset’ and reprogram the BM microenvironment in the hope that the tumors now become re-sensitized to therapies that they were resistant to previously.

## Figures and Tables

**Table 1:** Summary of newer single-agent treatment options that are being investigated for R/R DLBCL, FL and MCL.

Signaling Pathway/Mechanism Affected	Target	Drug	Complete and Partial Response Rate		
			DLBCL	FL	MCL
PI3K/AKT/mTOR	mTOR	Everolimus	30%	50%	32%
		Temsirolimus	36%	56%	22–40%
	AKT	MK2206	0%	25%	9%
	PI3K	Idelalisib	NA	57%	40%
		TGR-1202	11%	42%	33%
		Duvelisib	0%	67%	67%
		Copanlisib	25%	40%	71%
		Buparlisib	12%	25%	23%
	PI3K+histone deactylase	Fimepinostat	37%	NA	NA
B-cell receptor	SYK	Fostamatinib	22%	10%	11%
	BTK	Ibrutinib	28%	28%	68%–72%
		Acalabrutinib	NA	NA	81%
Apoptosis	BCL2	Venetoclax	18%	28%	75%
	Multiple targets	Lenalidomide	28%	NA	28%–40%
	Proteasome	Bortezomib	0%	NA	32%–41%
Immune checkpoint	PD1	Nivolumab	36%	40%	NA
	CD79b	Polatuzumab-vedotin	45%	NA	NA
	CD19	MOR208	26%	NA	NA
		Blinatumomab	43%	NA	NA

NA = Not Applicable. Table constructed with data from reference [80] and additional data from the latest available updates [81,82,83].

**Table 2:** CD-19 CAR-T cell-based therapies in R/R B-cell NHL.

Title	Axicabtagene Ciloleucel	Axicabtagene Ciloleucel	Tisagenlecleucel	Lisocabtagene Maraleucel
Clinical Trial	NCT02348216 (ZUMA-1)	NCT02601313 (ZUMA-2)	NCT02445248 (JULIET)	NCT02631044 (TRANSCEND)
Response Rate	ORR = 82% CR = 54%	ORR = 93% CR = 67%	ORR = 59% CR = 43%	ORR = 74% CR = 52%
Histological subtype (*n*)	DLBCL (77) tFL/PMBCL (24)	MCL (68)	DLBCL (51)	DLBCL (40) tDLBCL (14) FL grade 3B (1)

Abbreviations: ORR—overall response rate; CR—complete remission; DLBCL—diffuse large B-cell lymphoma; tFL—transformed follicular lymphoma; MCL—mantle cell lymphoma; tDLBCL—transformed DLBCL; R/R—relapsed/refractory. Table constructed with data from [125] and modified with latest updates.
